# Sleep-length differences are associated with altered longevity in the fruit fly *Drosophila melanogaster*

**DOI:** 10.1242/bio.054361

**Published:** 2020-09-16

**Authors:** Jacqueline B. Thompson, Oanh Oanh Su, Nou Yang, Johannes H. Bauer

**Affiliations:** Department of Chemistry, California State University, Sacramento, 6000 J Street, Sacramento, CA 95819, USA

**Keywords:** *Drosophila*, Aging, Sleep, Life span

## Abstract

Sleep deprivation has been shown to negatively impact health outcomes, leading to decreased immune responses, memory loss, increased activity of stress and inflammatory pathways, weight gain, and even behavioral changes. These observations suggest that sleep deprivation substantially interferes with important physiological functions, including metabolic pathways of energy utilization. Many of those phenotypes are correlated with age, suggesting that disrupted sleep may interfere with the aging process. However, little is known about how sleep disruption affects aging and longevity. Here, we investigate this relationship using eight representative fruit fly lines from the Sleep Inbred Panel (SIP). The SIP consists of 39 inbred lines that display extreme short- and long-sleep patterns, and constitutes a crucial *Drosophila* community resource for investigating the mechanisms of sleep regulation. Our data show that flies with short-sleep periods have ∼16% longer life span, as well as reduced aging rate, compared to flies with long-sleep. In contrast, disrupting normal circadian rhythm reduces fly longevity. Short-sleep SIP flies moreover show slight metabolic differences to long-sleep lines, and to flies with disrupted circadian rhythm. These data suggest that the inbred SIP lines engage sleep mechanisms that are distinct from the circadian clock system.

## Abstract (precis)

**Summary:** We show that fruit flies with shortened sleep patterns have decreased aging rates and increased longevity, while flies with disrupted circadian rhythm have decreased longevity. Our data suggests that circadian and sleep systems interact differentially with mechanisms of aging.

## INTRODUCTION

The circadian system is responsible for controlling daily activity patterns, including sleep (Jagannath et al., 2017; Laposky et al., 2005). Disruption of circadian rhythm has been shown to lead to metabolic dysregulation (Froy, 2011; Barclay et al., 2012), including Type-2 diabetes (T2D) (Marcheva et al., 2010; Shan et al., 2018; Pan et al., 2011) and insulin-sensitivity (Coomans et al., 2013). Furthermore, sleep and circadian disruption may cause behavioral changes and psychiatric disorders (Wulff et al., 2010; Jagannath et al., 2013).

Sleep-deficiency has long been known to negatively affect health outcomes (Depner et al., 2014). Even modest sleep-restriction has been shown to reduce insulin-sensitivity (Buxton et al., 2010), impair psychomotor function, and increase the secretion of pro-inflammatory cytokines (Vgontzas et al., 2004). Severe sleep reduction increases the risk for T2D (Cappuccio et al., 2010), depression, signs of cardiovascular disease and poor health (Newman et al., 1997; Pilcher et al., 1997; Cappuccio et al., 2008). On a molecular level, sleep reduction is associated with impaired insulin signaling and decreased glucose tolerance, increased nighttime cortisol and epinephrine levels (Spiegel et al., 1999; Nedeltcheva et al., 2009; Broussard et al., 2012; Guyon et al., 2014), and affects leptin levels and endocrine signaling (Spiegel et al., 2004; Vgontzas et al., 1999). Together, these effects lead to changes in metabolic energy balance (Calvin et al., 2013; Spiegel et al., 1999), and may result in obesity and T2D (Depner et al., 2014).

The first circadian clock genes were discovered in the fruit fly *Drosophila melanogaster* (Konopka and Benzer, 1971). Deletion of circadian clock-related genes leads to accelerated aging phenotypes, while overexpression of clock genes increases health parameters (Kondratov et al., 2006; Krishnan et al., 2009). In *Drosophila*, mutations in the gene *hyperkinetic* (*Hk*) (Bushey et al., 2010) or knockdown of the Jun-terminal kinase (JNK) pathway result in shortened sleep duration and reduced longevity (Takahama et al., 2012).

A possible connection point between the function of the circadian clock and metabolism are the sirtuins, a class of NAD-dependent histone deacetylases. It has been shown in mice that SIRT1 regulates the acetylation of Bmal1 and Per2, key components of the mammalian clock system (Nakahata et al., 2008; Asher et al., 2008; Wang et al., 2016). Interestingly, SIRT1 knockouts show accelerated aging phenotypes (Chang and Guarente, 2013), while SIRT1 overexpression increases longevity (Satoh et al., 2013) in mice, as does overexpression of dSir2 in *Drosophila* (Rogina and Helfand, 2004; Whitaker et al., 2013).

With increasing age, the function of the circadian clock system deteriorates (Wyse and Coogan, 2010; Kolker et al., 2003), possibly due to the accumulation of oxidative damage (Koh et al., 2006), in turn negatively affecting sleep and health outcomes (Hood and Amir, 2017). Interestingly, not only are sirtuins (Nakahata et al., 2008; Asher et al., 2008) involved in modulation of circadian signaling, but other aging-related pathways, such as AMPK (Lamia et al., 2009) or TOR activation (Cao et al., 2008) additionally modulate components of the circadian system, indicating that significant crosstalk exits between pathways of aging regulation and the circadian system.

Together, these data indicate a connection between the circadian system, metabolism and signaling pathways that modulate aging and longevity. While sleep disruption clearly negatively affects health outcomes, and possibly longevity, it is unclear whether improved sleep will benefit aging rates, and may thus lead to increased longevity.

To address this question, we made use of various *D. melanogaster* lines from the *Drosophila* Sleep Inbred Panel (SIP). The SIP consists of fruit fly lines that were specifically selected for short- and long sleep patterns (Serrano Negron et al., 2018) using an iterative breeding/selection approach. This unique resource thus consists of 39 distinct isogenic lines that only differ in the length of their sleep. An earlier version of the SIP has been used to identify 80 candidate genes with potential roles in sleep regulation (Harbison et al., 2017), highlighting the potential of the SIP for the dissection of the molecular basis of sleep.

Here, we investigate the connection between sleep length and aging by measuring the longevity and aging rates of eight representative SIP fly lines with short and long sleep patterns. In addition, we measure metabolic parameters to determine whether SIP fly display metabolic abnormalities.

## RESULTS

### SIP lines show consistent activity patterns

**Fig. 1.**
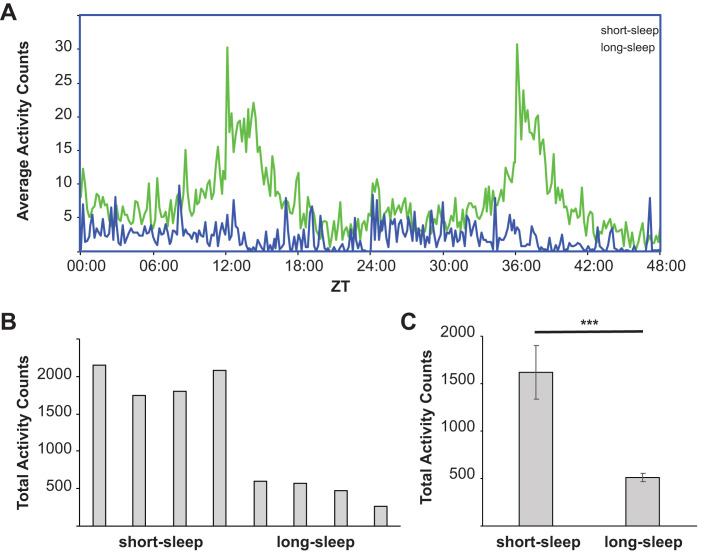
**Female activity pattern differences between SS and LS fly lines.** (A) Activity patterns of a representative SS line (green) and a representative LS line (blue) were observed over a 48 h recording interval. Shown is the average activity of at least eight separate recordings of a single female fly. (B) Total activity was integrated over a 48 h time period for four different SS lines and four different LS lines, using at least eight independent recordings per line. A representative experiment is shown. (C) Total activity counts over a 48 h period for each of the four SS and four LS, respectively, lines were combined. Shown are the average activity counts observed in three independent experiments (error bars represent the standard deviation, *P*=0.0026).

**Fig. 2.**
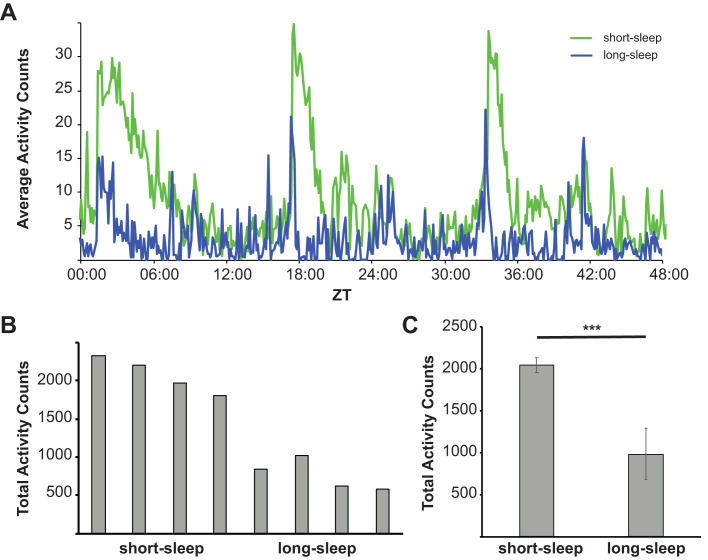
**Male activity pattern differences between SS and LS fly lines.** (A) Activity patterns of a representative SS line (green) and a representative LS line (blue) were observed over a 48 h recording interval. Shown is the average activity of at least eight separate recordings of a single male fly. (B) Total activity was integrated over a 48 h time period for four different SS lines and four different LS lines, using at least eight replicate recordings per line. (C) Total activity counts over a 48 h period for each of the four SS and four LS, respectively, lines were combined. Shown are the average activity counts observed in three independent experiments (error bars represent the standard deviation, *P*=0.0026).

In order to investigate the relationship between sleep length and aging, we obtained four representative lines each of short-sleep (SS) and long-sleep (LS) flies from the SIP. First, we confirmed that reported activity patterns still held after several years of propagation by measuring fly activity patterns over a 48 h time period. Individual female SS flies had greatly increased activity compared to LS flies (Fig. 1A). Increased activity of SS flies occurred mainly during the afternoon and early evening hours, with some additional increased activity occurring during night periods as described previously (Serrano Negron et al., 2018). Similar results were obtained in male flies (Fig. 2A).

Next, we quantitated the total activity of eight individual flies per line over the 48 h observation period (Figs 1B and 2B, for females and males, respectively). Total activity counts confirmed the SS versus LS differences, and, importantly, stayed consistent with little variation within their own cohort. Since each line may potentially represent a different genetic background, we subsequently combined the data from all four LS and SS lines, respectively, to obtain the average total activity count of LS versus SS flies. As shown Figs 1C and 2C, respectively, both female and male SS flies have significantly increased activity counts compared to LS flies.

### SS SIP flies have delayed aging rates

**Fig. 3.**
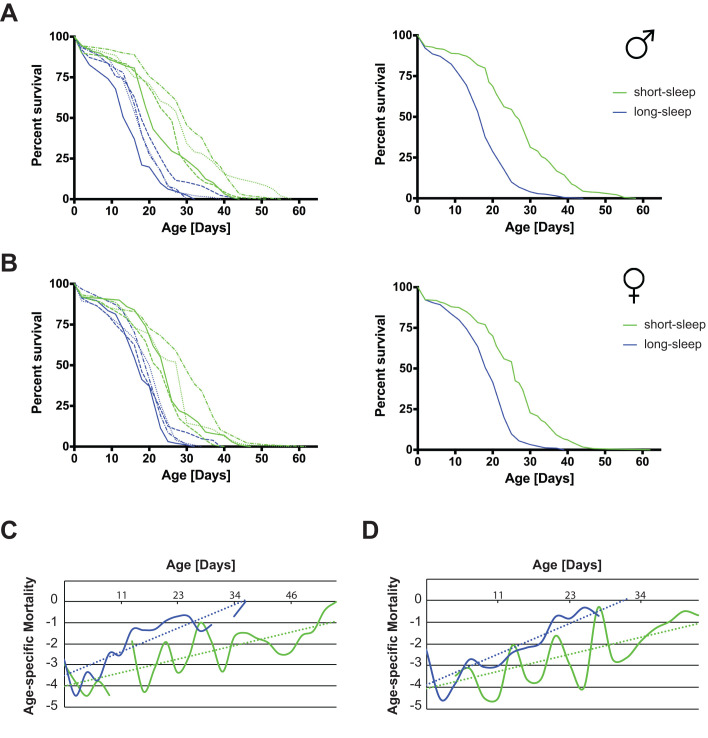
**Sleep length is associated with changes in longevity and age-specific mortality rates.** (A) Male and (B) female survivorship curves of four LS (blue) and four SS (green) *Drosophila* strains show increased longevity in the SS lines. The left panels show a representative of three independent experiments. Right panels show survivorship curves when all four lines each are combined. Average longevity and statistical analysis are shown in Tables 1 and 2. (C) Male and (D) female survivorship curves were converted into Gompertz mortality curves. Shown are one representative strain each of the SS (green) and the LS (blue) flies. The dotted lines represent the linear regression of the age-specific mortality rates. SS flies have a dramatic decrease in the slope of the mortality rate curve.

The activity data confirms that sleep phenotypes were retained even after several years of culture. Importantly, our data moreover validates that fly genetic background diverged only minimally since line inception, and that therefore all SIP lines can be considered iso-genic. Next, we measured the longevity of the eight SIP lines to determine whether sleep length is correlated with longevity. Interestingly, longevity is increased for both male (Fig. 3A) and female flies (Fig. 3B) in all four SS lines compared to the four LS lines (left panels, and Table 1). Combining all four SS and LS lines, respectively, into single survivorship curves to take the inter-line variation into account, similarly yields a statistically significant longevity increase of SS lines (Fig. 3A and B, right panels, and Table 2). When representative survivorship curves are converted into Gompertz mortality rate curves, a clear change in the slope of the linear regression is observed, while the Y-intercept remains largely unchanged (Fig. 3C for males and D for females). These data indicate that SS flies have a lower age-specific mortality rate, and therefore age slower than their LS counterparts.

**Table 1.**
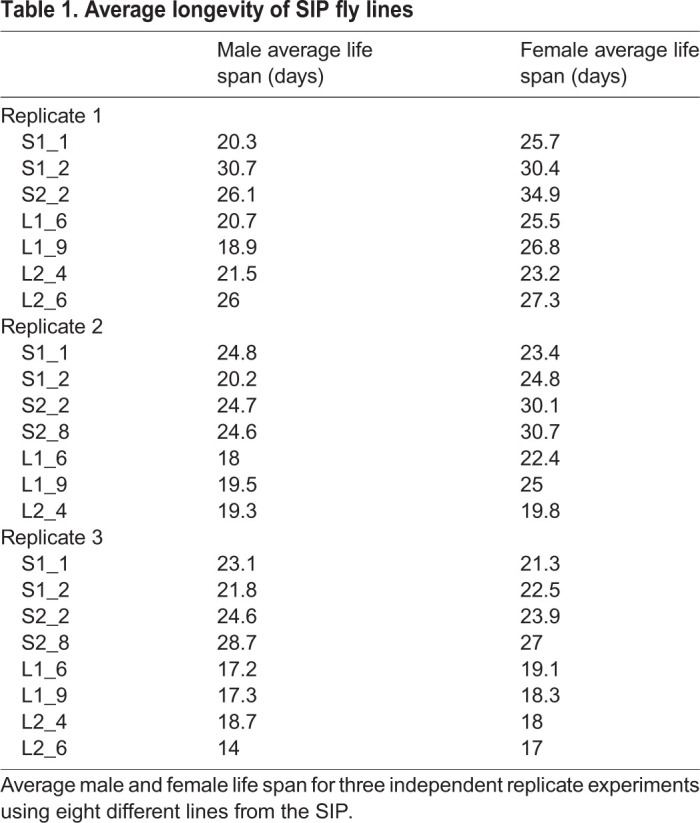
**Average longevity of SIP fly lines**

**Table 2.**
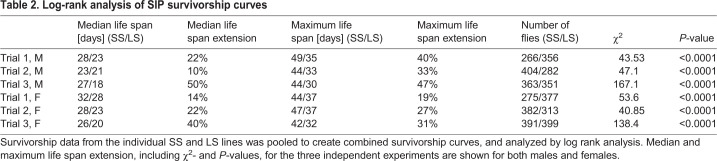
**Log-rank analysis of SIP survivorship curves**

### Disruption of circadian rhythm decreases longevity

**Fig. 4.**
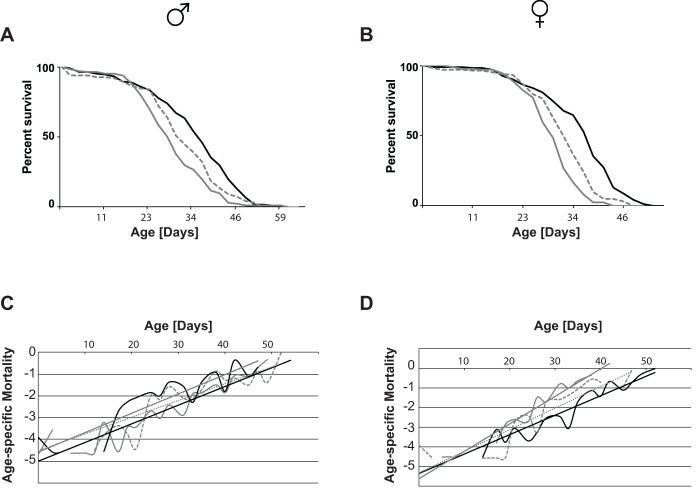
**Disruption of circadian rhythm alters longevity, but not mortality rates, in Canton-S wild-type flies.** (A,B) Flies raised under the standard 12 h light/dark cycle show longest life span (black), while disruption of the circadian pattern reduces longevity. Flies raised under constant light conditions show a ∼20% reduction in longevity (grey), while flies raised in constant darkness (grey dotted) have ∼12% reduced longevity (Table 3). Shown is a representative of three independent experiments [except for the constant dark experiment; similar results are observed in another commonly used laboratory strain w^1118^ (data not shown and Table 3)]. (C,D) Survivorship curves were converted into Gompertz mortality curves, and fitted to linear regression. In males, the slope of the mortality rate curve does not change between treatments, while in females a slight slope increase is observed under constant light conditions (black, 12 h light/dark; grey, constant light; grey dotted, constant dark).

These results are unexpected since we hypothesized that shortened sleep length leads to decreased health and longevity. We next investigated another paradigm of altered sleep by disrupting normal circadian rhythm, and raised flies under constant darkness or constant light. Under these conditions, fly longevity is reduced compared to flies raised under standard 12 h light/dark cycles (Fig. 4A, males; B, females). Conversion of survivorship curves into mortality rate curves shows no difference in the rate of aging compared to control treatment for male flies. Instead, a slight increase in the Y-intercept for flies with disrupted circadian rhythm is observed, which represents an increase in the external hazard function, rather than increased aging rate (Fig. 4C). In contrast, female flies with disrupted circadian rhythm had an increased slope, and thus an increase in the rate of aging, compared to control flies with normal circadian rhythm (Fig. 4D). Together, these results indicate that the mechanisms that modulate aging in response to circadian activity are different from those observed in long-lived SIP flies.

**Table 3.**
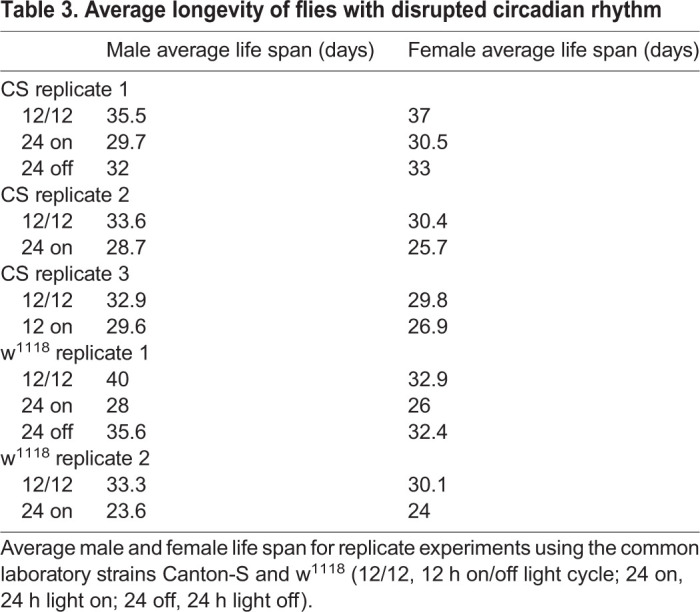
**Average longevity of flies with disrupted circadian rhythm**

### No gross metabolic abnormalities in SS SIP flies

**Fig. 5.**
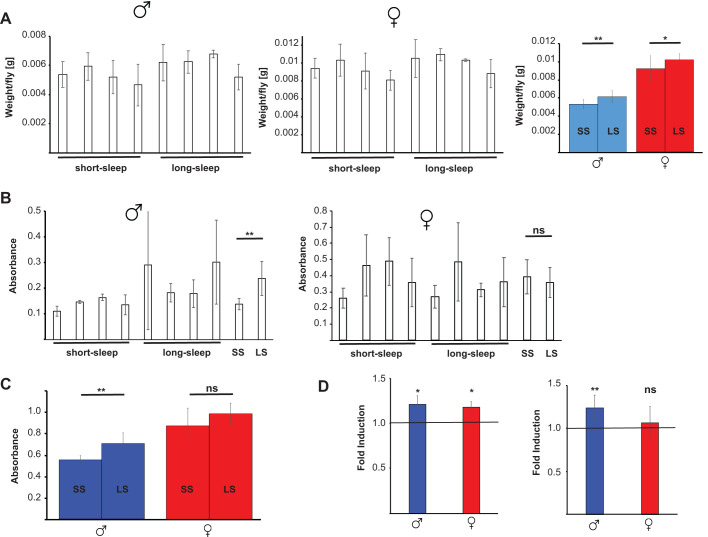
**Metabolic status of sleep-disrupted flies.** Flies were raised until 10 days of age, weighed and assayed for fat and glucose content. (A) Weight per fly of the four individual lines each with SS and LS, respectively, is shown in the left two panels. Averages of all lines is shown in the right panel. (B) Average absorbances at 550 nm of three independent experiments each are shown for the four SS and four LS lines, along with the averages of all lines. (C) Average absorbances of all SS and LS lines at 340 nm is shown for three independent experiments. (D) Fat (left panel) and glucose (right panel) measurements of flies raised under constant light conditions; data was normalized against results obtained with flies raised under standard light/dark conditions. Error bars represent the standard deviation of at least three independent experiments (SS, short-sleep; LS, long-sleep; ns, not significant; *P<0.05).

Increased activity is often associated with decreased levels of energy storage molecules. We therefore analyzed the metabolic status of the SIP lines by measuring weight, fat and glucose content. As shown in Fig. 5A, weight varies between the individual SS and LS lines. When the weights of all lines are averaged, a small, but significant, decrease in the weight of the SS lines is detected compared to LS flies. This difference is observed in both males and females.

Next, we investigated the amount triacylglycerides present in SS and LS flies. As with weight, some variation is observed between individual SS and LS lines. After averaging, long-lived male SS flies show a ∼10% reduction in fat content, while an analogous reduction is not observed in female flies (Fig. 5B). Similar results are obtained when glucose content is determined (Fig. 5C). Male SS flies have ∼10% reduction in glucose content, while females show no differences between SS and LS flies.

In contrast, short-lived flies with disrupted circadian rhythm due to constant light exposure have increased fat storage levels, as well as increased glucose content in males, but not in females (Fig. 5D). These data suggest that shortened longevity is associated with small, but significantly increased energy storage levels in male flies, but not female flies, and further highlights the mechanistic differences of the two sleep deprivation models.

## DISCUSSION

The SIP was created from the Sleep Advanced Intercross Population (SAIP) (Harbison et al., 2017) by selective breeding of LS and SS, respectively, followed by maintenance of lines for a total of 51 generations. At this point, 39 distinct lines were created by single fly mating (Serrano Negron et al., 2018). From this panel, we analyzed four randomly chosen lines each with long and short sleep patterns for their longevity. Our results indicate that SS lines have increased longevity and decreased rate of aging.

Since we expected unchanged or even shortened longevity in the SS lines (Harbison et al., 2017), we performed detailed statistical analysis of our longevity results. We combined the data from four different SS and LS lines each into one data set in order to merge any potential genetic background differences and to increase variability. Even under these parameters, we observe highly statistically significant longevity increases of SS flies over LS flies (Table 2). Inter-experiment variability is low, as determined by comparable median longevity (Table 1). These data demonstrate that under our experimental conditions, SIP SS flies are longer-lived than their LS counterparts. This longevity increase is due to a decreased aging rate, and not to a decreased risk of dying due to some inherent frailty in the LS flies.

SIP flies were initially assessed for longevity at generation 14. In contrast to our results, these early populations showed no clear longevity differences between long and short sleepers (Harbison et al., 2017). The reasons for the discrepancies in results are unclear. We evaluated longevity after additional breeding and isogenization steps to create the 39 lines of the SIP had been conducted. This in turn may accentuate longevity differences between SS and LS flies, due to reduced genetic background variability that may otherwise mask small differences in longevity.

Another explanation may be found in the different methodology to assess life span. The most important differences are food source and mating status. Here, we used fully mated flies, and kept male and female flies in the same population cage for the entire length of the assay, instead of using virgin flies. Virginity is known to significantly extend *Drosophila* longevity, an effect that may mask other life span-modulating events.

In addition, we used SY media, rather than the rich Bloomington Standard Media (BSM) for longevity assays. Rich media, such as the BSM, are optimized for fecundity and are generally used for stock maintenance. For longevity analysis, usually more defined and less-rich media are preferred (Mair et al., 2005) in order to evaluate potential effects of diet on longevity. The influence of diet on life span is well documented, especially in the context of Calorie or Dietary Restriction, (CR or DR, respectively). Interestingly, when we conduct preliminary longevity experiments under low calorie conditions, SS flies have, on average, shorter life spans than LS flies (data not shown), suggesting that SS flies may experience aspects of CR under normal raising conditions, resulting in increased longevity. Further decreasing calorie intake may thus lead to starvation phenotypes and shortened life span.

Animals experiencing CR usually express heightened activity patterns, due to increased foraging activity (Duffy et al., 1997), which are observed in SS flies. In addition, CR leads to depletion of energy storage molecules, such as triacylglycerides or carbohydrates, and weight loss (Bertrand et al., 1980). SS females do not show weight loss or energy storage diminution, while SS males experience a small, but significant weight and TAG loss. While these effects are small and need more extensive verification, together these data suggest that the increased life span observed in SS flies may at least partially be mediated by pathways that are involved in the CR response. In support of this hypothesis is the observation that SS flies show a reduction in the age-specific mortality rate, similar to flies under CR. Thus, at least in some flies of the SIP, sleep deficit may be tightly coupled to pathways that modulate longevity extension. Interestingly, genetic analysis of SAIP lines identified several genes with a known role in longevity modulation (Harbison et al., 2017). These genes include components of the JNK signaling pathways (*upd1*, *hep*, *bsk*) (Wang et al., 2005), the WNT signaling pathway (*sgg*, *fz*, *egr*) (Kamo et al., 2016) and the Insulin/Insulin-like Growth Factor (IIS) signaling pathway (*sktl*, *foxo*) (Grewal, 2009). A mutation in the IIS was the first gene to be identified as an aging gene in the nematode *Caenorhabditis elegans* (Friedman and Johnson, 1988), and subsequent work has clearly established a role for IIS in aging and longevity in a variety of model organisms and humans (Bartke, 2005). The IIS therefore connects metabolic networks to mechanisms of longevity modulation. Together, these data suggest that SIP flies not only show abnormalities in circadian networks, but also in signaling pathways connected to aging, demonstrating sleep regulation itself may be connected to pathways of longevity modulation.

## MATERIALS AND METHODS

### Fly culture and strains

Flies were kept on standard cornmeal medium (41) in a humidity- (50%) and temperature-controlled (25°C) incubator with a 12 h on/off light cycle. Four SS (SIP_S1_1, SIP_S1_2, SIP_S2_2, and SIP_S1_8) and four LS lines (SIP_L1_6, SIP_L1_9, SIP_L2_4, SIP_L2_6) from the SIP were obtained from the Bloomington *Drosophila* Stock Center at Indiana University (Bloomington, IN, USA).

### Life span studies

For longevity analysis, flies were collected under light CO_2_ anesthesia within 24 h of eclosion, and housed at a density of 100 males and 100 females per population cage on 10% SY food (10% sucrose, 10% yeast extract). Fresh food was provided and dead flies were removed and scored for sex every 2–3 days.

For experiments with disrupted circadian rhythm, Canton-S and w^1118^ flies were kept under the same housing and food conditions, except for the length of the light cycle, which was set to 12 h on/off, 24 h on, and 24 h off, respectively.

### Sleep activity measurements

Sleep measurements were performed as previously described (Serrano Negron et al., 2018). Flies were collected and cultured as for longevity analysis, and aged for 10 days. Next, flies were separated by sex, and placed as single flies into DAM2 *Drosophila* Activity Monitor tubes (Trikinetics Inc.). After a 12 h adjustment period, measurements were taken over 48 h using eight animals each per trial. Activity was recorded in 10 min intervals.

### Determination of metabolite levels

Flies were raised as described above and aged for 10 days. Flies were anesthetized, weighed in groups of ten flies, and transferred to chilled microcentrifuge tubes.

Lipid analysis was performed as described (Hildebrandt et al., 2011) by adding 150 µl 0.05% TWEEN-20 solution, followed by homogenization using a motorized micro pestle. After centrifugation, supernatants were transferred to fresh tubes. 10 µl of supernatants were used with the Infinity Triacylglyceride Determination Kit (Thermo Fisher Scientific), incubated for 10 min at room temperature, and absorbances were recorded at 550 nm using a BioTek Synergy2 96-well plate reader.

For analysis of glucose levels, flies were homogenized in 300 µl 1× RIPA buffer, supplemented with 1 mM DTT, and homogenized as above. After centrifugation, supernatants were used with the Infinity Glucose Determination Kit (Thermo Fisher Scientific), incubated for 15 min at room temperature, and absorbances were recorded at 340 nm.

### Statistics

Statistical analyses, including log-rank and *t*-tests, were performed using the Prism suite of biostatistical software (GraphPad, San Diego, USA). Maximum life span was calculated as the median age of the last surviving 10% of the population.
